# Ghrelin Expression in the Mouse Pancreas Defines a Unique Multipotent Progenitor Population

**DOI:** 10.1371/journal.pone.0052026

**Published:** 2012-12-12

**Authors:** Luis Arnes, Jonathon T. Hill, Stefanie Gross, Mark A. Magnuson, Lori Sussel

**Affiliations:** 1 Department of Genetics and Development, Columbia University, New York, New York, United States of America; 2 Department of Molecular Physiology and Biophysics and Center for Stem Cell Biology, Vanderbilt University School of Medicine, Nashville, Tennessee, United States of America; University of British Columbia, Canada

## Abstract

Pancreatic islet cells provide the major source of counteractive endocrine hormones required for maintaining glucose homeostasis; severe health problems result when these cell types are insufficiently active or reduced in number. Therefore, the process of islet endocrine cell lineage allocation is critical to ensure there is a correct balance of islet cell types. There are four endocrine cell types within the adult islet, including the glucagon-producing alpha cells, insulin-producing beta cells, somatostatin-producing delta cells and pancreatic polypeptide-producing PP cells. A fifth islet cell type, the ghrelin-producing epsilon cells, is primarily found during gestational development. Although hormone expression is generally assumed to mark the final entry to a determined cell state, we demonstrate in this study that ghrelin-expressing epsilon cells within the mouse pancreas do not represent a terminally differentiated endocrine population. Ghrelin cells give rise to significant numbers of alpha and PP cells and rare beta cells in the adult islet. Furthermore, pancreatic ghrelin-producing cells are maintained in pancreata lacking the essential endocrine lineage regulator Neurogenin3, and retain the ability to contribute to cells within the pancreatic ductal and exocrine lineages. These results demonstrate that the islet ghrelin-expressing epsilon cells represent a multi-potent progenitor cell population that delineates a major subgrouping of the islet endocrine cell populations. These studies also provide evidence that many of hormone-producing cells within the adult islet represent heterogeneous populations based on their ontogeny, which could have broader implications on the regulation of islet cell ratios and their ability to effectively respond to fluctuations in the metabolic environment during development.

## Introduction

Pancreatic islets provide the major source of endocrine hormones required for maintaining glucose homeostasis. Adult islets are comprised of four endocrine cell types that have historically been defined by their hormone expression: glucagon (Gcg)-producing alpha cells, insulin (Ins)-producing beta cells, somatostatin (Sst)-producing delta cells and pancreatic polypeptide (Ppy)-producing PP cells. In mice, each mature islet endocrine population and a transient ghrelin (Ghrl)-expressing epsilon cell population are derived from Neurogenin3 (Neurog3)-expressing progenitor cells in a time-dependent manner during embryonic development [1], [2]. Although considerable research efforts have begun to elucidate the regulation of endocrine cell differentiation during embryogenesis (reviewed in [3], [4]), the lineage relationships between each endocrine population remain obscure. Furthermore, several recent studies have revealed previously unappreciated plasticity between the differentiated pancreatic cell populations during fetal development and in the adult [5]–[9]. Therefore, a greater understanding of the precise ontogeny and lineage relationships of the pancreatic endocrine and non-endocrine populations could aid efforts to generate and maintain functional beta cells from alternative cell sources for cell-based diabetes therapies.

During embryonic development, each of the peptide hormones, with the exception of Ppy, can be detected as early as embryonic day (E) 9.5 in the dorsal pancreas, with glucagon being the most abundant and somatostatin being quite rare [3]. At the earliest stages of development, ghrelin-expressing cells are also quite numerous and equivalent numbers of glucagon- and ghrelin-producing single positive cells are present by e11.5. Beginning around e12.5–e13.5, there is a major wave of endocrine cell differentiation called the secondary transition that significantly expands the alpha, beta and delta cell populations [3], [10]. Scattered single Ppy-positive PP cells can first be detected at e11.5 predominantly in the ventral pancreas, however significant numbers of PP cells are not evident until late gestation [3]. Although lineage relationships have been proposed between many of the endocrine cell populations based on co-expression studies and/or reciprocal changes in relative cell type numbers in genetically modified mice [11]–[14], genetic lineage analyses has not always confirmed these putative connections during normal development [15]. However, lineage relationships are likely to exist and there is increasing evidence that transdifferentiation can occur between certain islet cell populations, including alpha and beta cells, in modified genetic backgrounds [5]–[8]. Furthermore, several studies have demonstrated that the number of ghrelin cells is often inversely related to the other endocrine cell populations in genetically manipulated mice, suggesting a reciprocal lineage relationship exists between ghrelin cells and the other endocrine cell types [1], [16]–[18].

Ghrelin-producing cells are the most recently identified endocrine population in the pancreatic islet. Although the initial reports of ghrelin expression in the pancreas were somewhat conflicting [19]–[22], there is now strong evidence that ghrelin is expressed exclusively in the epsilon cells and a small subset of alpha cells in embryonic rodent and human islets [1], [16], [22], [23]. The mono-hormonal epsilon population represents approximately 5–10% of the total number of islet endocrine cells at birth, after which epsilon cell numbers gradually decline [22]–[24]. It is not known whether the epsilon cells undergo apoptosis, persist without producing hormones or differentiate into other cell types as the number of ghrelin-expressing cells decline perinatally. In this study, we used Cre-lox lineage analysis to determine the fate of all ghrelin-expressing cells during development and in the adult. These experiments unexpectedly revealed that the majority of ghrelin-producing epsilon cells contribute to almost half of the PP cell population in the adult islet. These studies also show that the glucagon/ghrelin co-expressing cell population is maintained postnatally, although ghrelin expression becomes extinguished and these cells ultimately represent approximately 25% of the mature alpha cell population. Finally, a population of ghrelin-expressing cells that form independently of the Neurog3+ endocrine lineage retain the ability to populate cells within the acinar and ductal lineages. Importantly, these findings demonstrate that a population of pancreatic endocrine cells activate hormone expression, but do not become terminally differentiated. Furthermore, we demonstrate a novel lineage relationship between pancreatic ghrelin-expressing cells and other endocrine cell populations to provide insight into the formation of distinct endocrine lineage subgroups. Notably these studies also reveal a previously unrecognized heterogeneity within the mature endocrine cell types based on their ontogeny that could have far-reaching implications regarding islet cell function and plasticity during development and in the adult.

## Materials and Methods

### Generation of Ghrelin RMCE ES cells and Ghrl:Cre-eGFP mice

**Figure 1:**
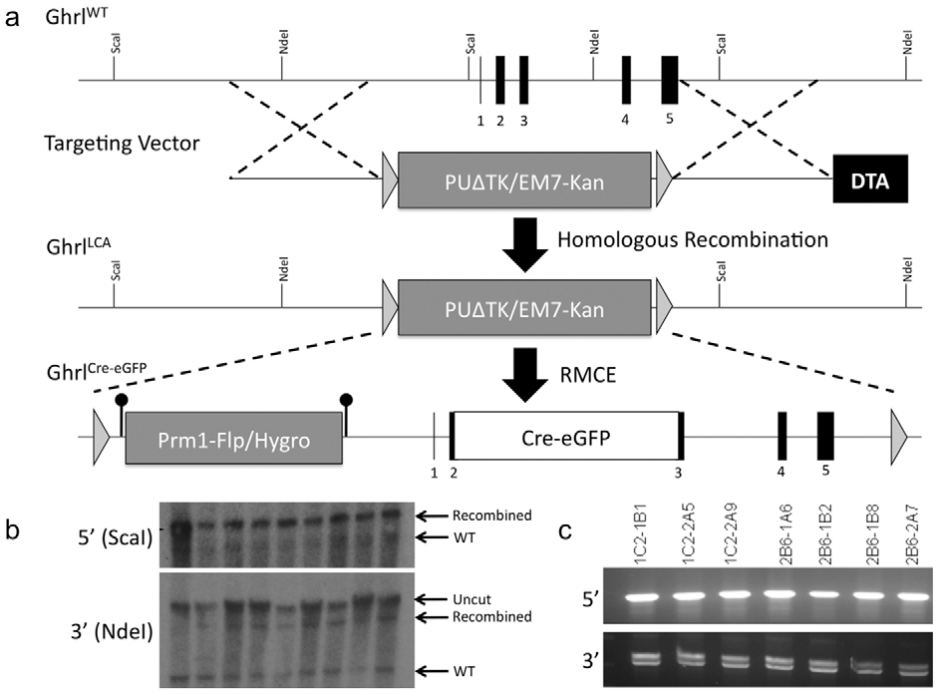
Construction of the Ghrl^LCA^ acceptor and Ghrl:Cre-eGFP exchange alleles. (a) Schematic of the Ghrl:Cre-eGFP knock-in allele generated using recombinase mediated cassette exchange (RMCE). First, a floxed Ghrelin (Ghrl^LCA^) allele was created by inserting a positive/negative selection cassette flanked by the Lox71 and Lox2272 modified lox sites into the ghrelin locus by homologous recombination. The Lox71 and Lox2272 sites allow for directional and irreversible Cre mediated recombination while suppressing intramolecular recombination. An exchange cassette vector containing the complimentary Lox66 and Lox2272 sites and a Cre-eGFP coding sequence spanning exons 2 and 3 (Ghrl:Cre-eGFP) was then created and inserted into the genome using RMCE. (b) Insertion of the Ghrl^LCA^ allele was confirmed by Southern blot using either ScaI digested (5′ recombination) or NdeI digested (3′ recombination) genomic DNA. (c) Correct exchange of the Ghrl:Cre-eGFP allele in clones that survived both positive and negative selection was confirmed by PCR analysis. All seven tested clones had correct allele exchange.

Ghrl:Cre-eGFP mice were generated using the RMCE protocol as described previously [25]. Schematic representation of the strategy is shown in Figure 1. Briefly, modified lox sites flanking positive and negative selection cassettes were inserted into the ghrelin locus spanning a 6912 bp region from 2678 bp upstream of exon 1 to 473 bp downstream of exon 5 using homologous recombination to create the Ghrl-LCA (locus cassette acceptor) allele in mouse embryonic stem cell (ES). Confirmation after positive selection was done by Southern blot analysis for both the 5′ (ScaI digestion. 19 kb fragment vs. 17 kb fragment) and 3′ arm (NdeI digestion. 18 kb fragment vs. 8 kb fragment). The Ghrl:Cre-eGFP allele was then created using recombineering to insert a Cre-eGFP coding region into a plasmid vector containing the region of the ghrelin locus removed in the Ghrl-LCA allele. The construct was designed to insert the Cre-eGFP open reading frame at the endogenous start codon. This plasmid was co-injected with a Cre-expressing vector into ES cells carrying the Ghrl-LCA allele. Cells were screened for loss of the selection cassette in the Ghrl-LCA allele and for hygromycin resistance. Surviving clones were then injected into blastocysts from natural matings of C57/BL6 mice. Resulting chimeras were mated to Swiss-Black mice (Taconic) and germline passage of the allele was confirmed by PCR using 5′-AGGCACCACATCCCCAGGCA-3′ and 5′-CCACGACCGGCAAACGGACA-3′. Removal of the FRT-flanked *HygroR* cassette was accomplished by inbreeding with *ACTB:FLPe* mice (Jackson).

### Animals

All mice were maintained on a Swiss Black (Taconic) background and housed and treated according to the Columbia University Institutional Animal Care and Use Committee approval protocol AAAC0259. Lineage tracing experiments were performed in *Ghrl* ^Cre-eGFP/+^ (G*hrl*-Cre); *Rosa26* ^Tom/+^ or *Rosa26* ^Tom/Tom^ (*R26*-TOM) in wild type and Nkx2.2 or Neurog3 knockout backgrounds. *Nkx2.2^+/−^* (Nkx2-2^tm1Jlr^; MGI:1932100) and *Neurog3^tTA/+^* (Neurog3^tm2(tTA)Ggu^; MGI:3850054) strains were described previously [26], [27]. *R26*-TOM (B6.Cg-*Gt(ROSA)26Sor^tm14(CAG-tdTomato)Hze^*/J) was obtained from the Jackson Laboratory.

### Tissue

Pancreata from P0 and 8-week-old mice, and stomach and intestine from 6-week-old mice were dissected from the animal and fixed in 4% PFA overnight at 4°C. Fixed tissue was washed in cold PBS and cryoprotected with 30% sucrose for 24 h. Tissue was embedded in O.C.T (Tissue Tek) and frozen at –80°C. 10 µm-thick sections spanning the entire organ were collected. 5 µm-thick sections were used for analysis of intestinal tissue.

### Immunofluorescence

Sections were blocked in the appropriate serum (5% serum in 1×PBS +0.5% Triton-X) for 30 minutes. Primary antibodies were diluted in blocking buffer and incubated on tissue sections overnight at 4°C. The primary antibodies used were: rabbit α-5-HT (stomach, 1∶500; intestine, 1∶200; Immunostar), rabbit α-amylase (1∶500; Sigma-Aldrich), rabbit α-chromogranin A (1∶500; Abcam), rabbit α-gastrin (1∶200; Phoenix Pharmaceuticals, Inc), goat α-ghrelin (pancreas and stomach, 1∶800; intestine, 1∶200; Santa Cruz Biotechnologies, Inc), guinea pig α-glucagon (1∶1000; Linco), guinea pig α-insulin (1∶200; Linco), rabbit α-insulin (1∶200; Cell Signaling Technology), rabbit α-pancreatic polypeptide (1∶500; Zymed), rabbit α-somatostatin (pancreas and stomach, 1∶500; intestine, 1;200; Phoenix Pharmaceuticals, Inc), rabbit α-substance P (1∶200, Phoenix Pharmaceuticals, Inc). Sections were incubated with appropriate secondary antibodies conjugated to DyLight-488, DyLight-549 and DyLight-649, Alexa-488, Alexa-647 (Jackson Immunoresearch). Fluorescein-Dolichos Biflorus Agglutinin (DBA, Vector Labs, Burlingame, CA, USA), when used, was incubated together with the primary antibodies. DAPI (1∶1000; Invitrogen) was applied for 30 minutes following secondary antibody incubation. Tomato signal was detected by direct fluorescence of the protein. Images were acquired with either an epifluorescence (Leica DM5500) or a confocal (Nikon A1R MP) microscope. Note that all panels showing “ghrelin” staining represents images of ghrelin immunoreactivity using primary anti-ghrelin antibody.

### Morphometric Analysis and Cell Counting

Morphometric analysis was performed in P0 and 8-week-old pancreas. The entire organ was sectioned and evenly distributed 10 µm sections were selected. Six sections in P0 pancreas and eight sections in adult pancreas were analyzed. Cre-recombination efficiency was determined by dividing the number of Ghrl^+^ and Tom^+^ cells by the total number of Ghrl^+^ cells in P0 pancreas. Total number of Tom^+^ cells within the endocrine compartment was assessed by dividing the number of Tom^+^ cells in the endocrine compartment by the total endocrine area measured by chromogranin A^+^ staining. Total number of Ghrl^+^ cells in Neurog3^tTA/tTA^ was determined by dividing the number of Ghrl^+^ cells by the total pancreatic exocrine area as defined by amylase staining. Endocrine and exocrine area was quantified using Image Pro Plus 5.0.1 software (Media Cybernetics).

## Results

### Generation and Characterization of Ghrelin:Cre-eGFP mice

**Figure 2:**
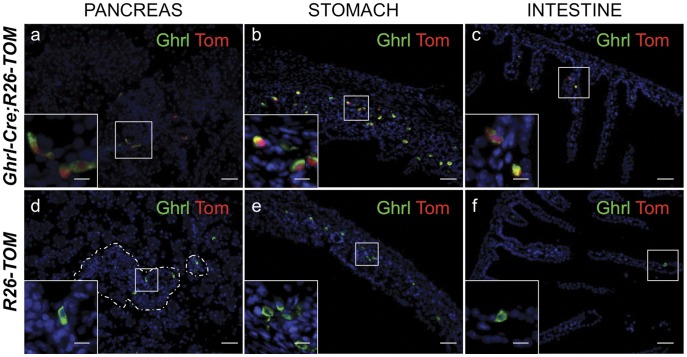
Reporter expression accurately recapitulates ghrelin expression at e18.5. Tom+ cells co-express ghrelin in pancreas (a), stomach (b) and duodenum (c). Note that the pancreatic ghrelin-producing epsilon population only represent 5–10% of the total pancreatic endocrine population at this stage of development. No leakiness of the reporter was detected in the absence of the *Ghrl:Cre* allele in any of the tissues studied (d–f). Scale bar, all panels 50 µm, insets 20 µm.

To determine the fate of ghrelin-producing islet epsilon cells during embryonic development and adulthood, we generated mice containing a Cre-eGFP fusion protein knocked into the ghrelin locus using recombination mediated cassette exchange (RMCE) technology [25], [28], [29] (Figure 1; Materials and methods). The resulting *ghrelin^Cre-EGFP/+^* mice were crossed with a *ROSA26-Tomato* (*R26^Tom/+^* or *R26^Tom/Tom^*) mouse reporter line to lineage trace the ghrelin-expressing cells during development and in the adult [30]. Mice containing either one or two copies of the *ghrelin^Cre-EGFP^* allele were euglycemic and did not display defects in pancreas development or islet cell lineage formation (data not shown), consistent with previous characterization of ghrelin null mice [31]–[33]. There was also no apparent difference in the lineage tracing outcomes when the reporter was present in the heterozygous versus homozygous state. At P0, the R26-Tomato reporter co-localized with ghrelin in 60% of the pancreatic epsilon cells and in 60% of the X/A cells of the stomach (Figure 2). The reporter was also detected in 30–50% of ghrelin cells in the duodenum, where ghrelin is expressed at lower levels (Figure 2). We did not detect tomato reporter expression in the absence of the *ghrelin^Cre-EGFP^* allele or in ghrelin non-expressing tissues, indicating the specificity and fidelity of the Cre lineage reporter system in these mice (Figure 2d–f). Although the Cre was highly active, we were unable to detect eGFP expression in any ghrelin-expressing tissues (figure S1), therefore all ghrelin expression analyses described in this study were performed with indirect immunofluorescence (see materials and methods).

**Figure 3:**
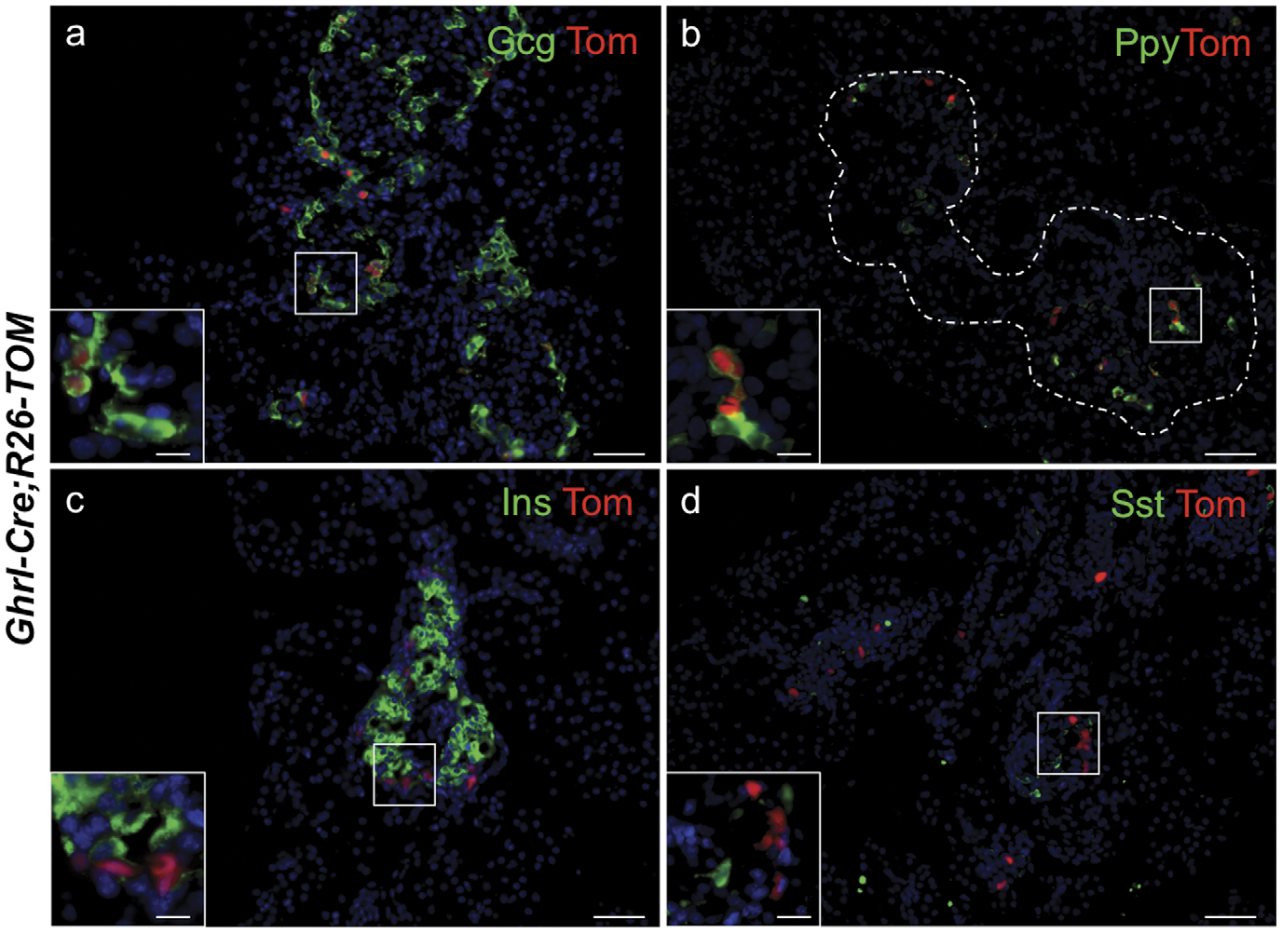
Ghrelin-labeled cells mark a subpopulation of glucagon- and Ppy-expressing cells in neonates. Representative images showing immunofluorescence of islet hormones and direct tomato reporter fluorescence on cryosections of *Ghrl-Cre;R26-Tomato (TOM)* P0 pancreas (a-d). Reporter fluorescence co-localized with a significant number of glucagon-expressing cells (a) and Ppy-expressing cells (b). No significant co-localization was detected with insulin- (c) and somatostatin-expressing cells (d). DAPI staining was used to visualize the nuclei. Scale bars: low magnification = 50 µm, insets = 10 µm.

Previously published expression studies described the existence of single hormone ghrelin-producing epsilon cells and ghrelin+ glucagon+ co-expressing cells in the neonatal mouse islet [3], [16]. Consistent with the co-expression studies, ghrelin cell lineage analysis at P0 demonstrated that the majority of tomato-positive cells expressed ghrelin (43%), glucagon (31%) and ghrelin and glucagon (20%) (Figure 3; Figure S2a–b) [1], [3], [16]. We also observed 5% of Ppy-expressing cells that co-express the tomato lineage label (Figure 3b), which is also consistent with previous hormone co-expression studies [1], [22]. We did not observe R26-Tomato reporter expression in insulin-expressing beta cells or somatostatin-expressing delta cells at birth (Figure 3c–d). Furthermore, consistent with the absence of hormone expression in the Neurog3 precursor cells [34]–[37], we also did not detect reporter expression in the Neurog3+ cell population (Figure S2c).

### Ghrelin Lineage-labeled cells Persist in the Adult and Give Rise to Non-ghrelin-producing Endocrine Cells

**Figure 4:**
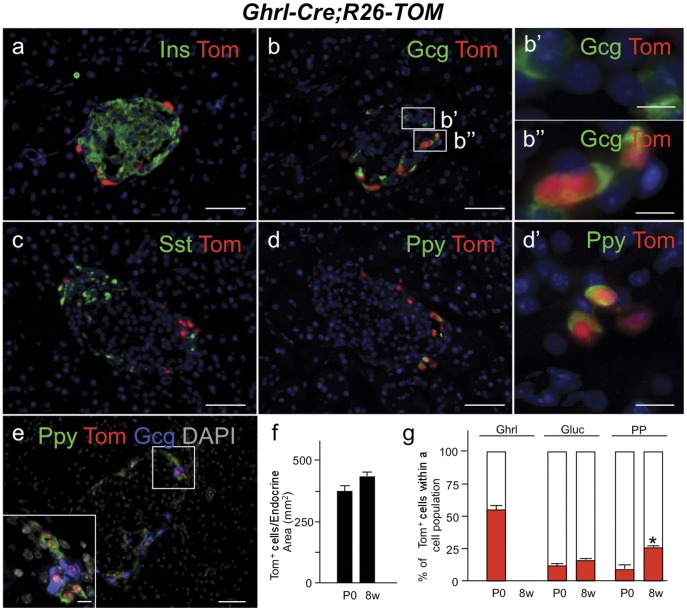
Two distinct populations of alpha and PP cells can be distinguished in the adult endocrine pancreas based on their origin. We analyzed pancreata from 8 week old (8w) *Ghrl-Cre;R26-TOM* mice. In the adult, we could not detect ghrelin expression by immunofluorescence but ghrelin-lineage labeled cells localized primarily to the periphery of the islet. (a–b) Adjacent images of the same islet of Langerhans showing co-localization of the ghrelin-lineage labeled cells with glucagon (b) but not with insulin (a). White boxes in b shows two distinct population of alpha cells, one derived from a ghrelin-expressing cell (magnified in b”) and one independent of the ghrelin lineage (magnified in b). (c–d) Adjacent images show that ghrelin-expressing cells contribute to the PP (magnified in d’) but not to the delta lineage. (e) Immunofluorescence analysis with glucagon and Ppy antibodies confirm that two independent populations of alpha and PP cells are derived from ghrelin-expressing cells. (f) Quantitative analysis of the total number of ghrelin-labeled cells per endocrine area at the two stages studied, P0 and 8w pancreas. (g) Fraction of ghrelin-labeled cells within a given population of hormone producing cells at P0 and 8w. The percentage of ghrelin cells labeled with the reporter at P0 is indicative of the Cre recombination efficiency. No ghrelin cells were detected at 8w. Three mice were examined per time point. Error bars show mean ± SEM (p<0.05 (*) Student’s unpaired t-test). DAPI staining was used to visualize the nuclei. Scale bar: low magnification = 50 µm, B, B’ and inset in E = 10 µm, d’ = 20 µm.

In the wild-type pancreatic islet, the number of ghrelin-expressing epsilon cells peaks at birth and declines postnatally; very few ghrelin+ cells can be found in the adult islet [3], [23], [24]. Interestingly, quantification of the tomato-expressing cells in neonatal and 8 week old islets suggested that equivalent numbers of ghrelin-descendants are maintained in the adult islet (Figure 4f). To determine the fate of the epsilon cells in the adult, we performed co-expression analysis with the R26-Tomato lineage reporter and each islet endocrine hormone. Interestingly, approximately 14% of the glucagon-expressing alpha cells in the adult islet are labeled with the tomato reporter, suggesting that the embryonic glucagon/ghrelin co-labeled population is maintained in the adult, although ghrelin expression is extinguished (Figure 4b and g). Over 25% of the adult PP cells are also labeled with the tomato reporter (Figure 4d and g). The increase in ghrelin-lineage labeled PP cells postnatally is concomitant with the decrease in ghrelin-expressing mono-hormonal cells, suggesting that the majority of epsilon cells are giving rise to a PP subpopulation (Figure 4d, d’ and g; Figure S3a–b). Since the Cre-mediated labeling efficiency of ghrelin cells was approximately 60%, it is likely that the number of lineage-labeled cells is underestimated, and the extrapolated number of ghrelin-lineage labeled alpha and PP cells may represent as much as 30% and 50%, respectively, of the adult populations. The ghrelin-derived alpha and PP cell populations appear to represent distinct populations, as we do not detect co-expression of glucagon and Ppy in the tomato-positive lineages (Figure 4e). Furthermore, the majority of lineage-labeled alpha cells were present in the dorsal pancreas, whereas the lineage-labeled PP cells were located in the ventral pancreas. Although most beta cells were not descendants of the epsilon cells (Figure 4a), rare tomato and insulin co-expressing cells could be detected in the adult islet (figure S3d). We did not detect tomato expression in somatostatin-expressing delta cells, suggesting that epsilon cells do not contribute to the delta cell lineage (Figure 4c). Cumulatively, these data indicate that the pancreatic ghrelin-expressing cells contribute to major subpopulations of mature alpha and PP cells and rare beta cells in the adult islet, indicating the existence heterogeneous endocrine subpopulations based on their ontogeny.

### Ghrelin Descendants Give Rise to Ductal and Exocrine Lineages

**Figure 5:**
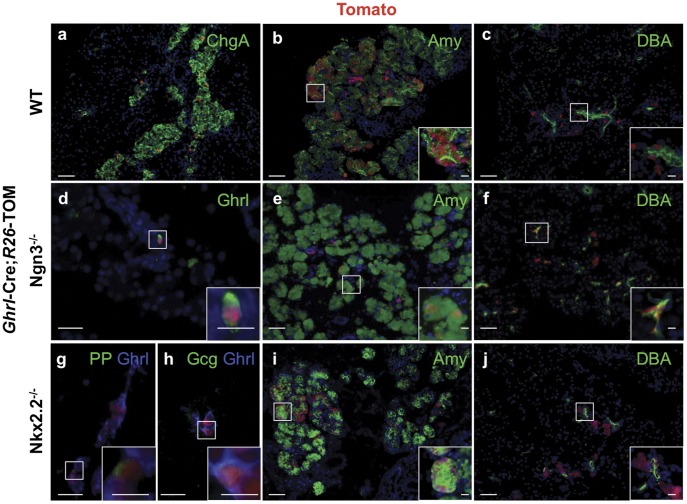
Ghrelin lineage-labeled cells are frequently found in the pancreatic exocrine and ductal compartments in neonates. Ghrelin-lineage labeled Tom+ cells predominantly localize to the islet periphery (a). Approximately 50% of the embryos analyzed contained ghrelin-labeled cells within the amylase+ acini clusters (b). This phenomenon is preferentially observed in juxta-duodenal regions of the ventral pancreas. (c) Ghrelin-lineage labeled cells that co-expressed ductal markers were also identified in the same animals. In Neurog3^−/−^ pancreata (d–f), which are almost completely devoid of endocrine hormone-producing cells, we could detect a small number of ghrelin-expressing and lineage labeled cells (d). We also detected ghrelin descendants in the exocrine (e) and ductal tissue (f) of Neurog3^−/−^ embryos. (g–j) In Nkx2.2^−/−^ mice, a subset of Ppy- (g) and glucagon-expressing cells (h) are still derived from ghrelin-expressing cells. Ghrelin-labeled cells are also detected in the exocrine and ductal compartment of Nkx2.2^−/−^ animals (i–j). The frequency and magnitude of lineage labeling of these cells were not significantly different from wild type pancreas. a, d and e are representative images of at least 3 pancreata. b, c, f, g, h, i, j are representative image of at least 3 embryos in which we detected labeled cells localized to the exocrine and ductal pancreatic tissue. All images are from *Ghrl-Cre;R26-TOM* P0 pancreas in the indicated genetic backgrounds. DAPI staining was used to visualize the nuclei. Scale bar: all panels = 50 µm, insets = 10 µm.

Although the majority of ghrelin lineage-labeled cells were located in the endocrine compartment, we also detected ghrelin-descendants in the ductal and exocrine tissues of neonatal mice (Figure 5a–c). Ghrelin descendants localized to the epithelial ducts were positive for DBA lectin (Figure 5c). In the majority of animals analyzed, 5% of the exocrine tissue appears to be derived from ghrelin-expressing cells, resulting in clusters of labeled acini in the developing embryo and in the adult (Figure 5b, figure S4). Interestingly, ghrelin lineage-labeled acini appeared more frequently in the ventral pancreas.

### A Population of Epsilon Cells is Independent of Neurog3 and Nkx2.2 Function

Previous studies have shown that the majority of epsilon cells are derived from the Neurog3 endocrine progenitor population [1]. Consistently, the ghrelin lineage-labeled alpha and PP cells are absent in neonatal Neurog3 null mice. Since ghrelin-derived progeny also appear to contribute to exocrine and ductal lineages (Figure 5), we hypothesized that a Neurog3-independent ghrelin-expressing, multi-potent cell population exists. Supporting this hypothesis, we identified a small number of ghrelin-expressing cells in the pancreas of *Neurog3* null mice (2 cells/mm^2^ P0 pancreas; Figure 5d). Furthermore, in the absence of Neurog3, ghrelin-expressing cells are still able to contribute to 5% of the exocrine and ductal lineages (Figure 5e–f), suggesting that ghrelin-expressing cells represent a unique Neurog3-independent cell lineage with the potential to contribute to both endocrine and non-endocrine lineages.

In the pancreas of *Nkx2.2* null embryos, beta cells are absent, alpha cells and PP cells are reduced, and the ghrelin-expressing population represents over 90% of the islet endocrine cells and display increased ghrelin expression per cell; this increased expression pattern is maintained throughout gestation (figure S5) [16]. Consistent with the higher ghrelin expression levels, Cre is active in 90% of the ghrelin cells in *Nkx2.2* null pancreata (Figure S5). Despite the increased Cre activity and significant reduction in the numbers of PP and alpha cells in the *Nkx2.2* null mice [16], the relative number of PP and alpha cells derived from the ghrelin-expressing population is not altered (Figure 5g–h). This would suggest that the ghrelin-derived PP and alpha cell populations arise independently of Nkx2.2 function. Furthermore, despite the higher propensity to express ghrelin throughout the P0 *Nkx2.2* null pancreas, we also do not detect a significant change in the relative ratios of ductal and exocrine lineages arising from the expanded ghrelin population (Figure 5i, j), suggesting that the labeling of non-ghrelin lineages is not due to spurious ghrelin promoter activation in non-epsilon cells and supports the existence of a defined Nkx2.2-independent ghrelin multi-potent progenitor that can give rise to endocrine, exocrine and ductal cells.

### Intestinal and Stomach Ghrelin-labeled Cells are Restricted to Endocrine Lineages

**Figure 6:**
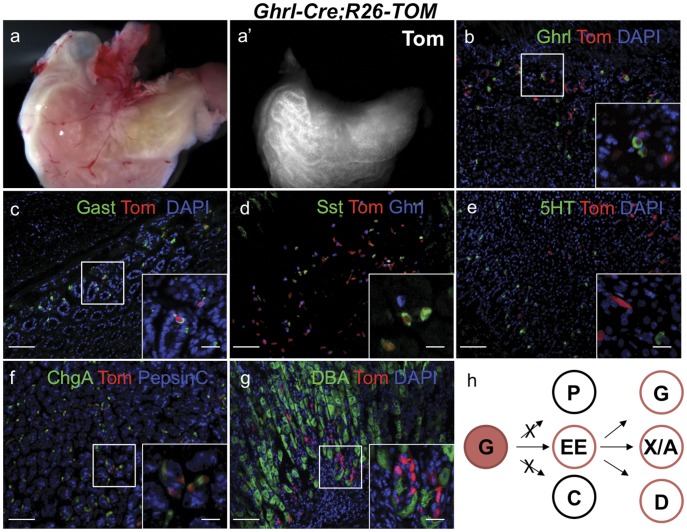
Ghrelin lineage-labeled cells are committed to the endocrine lineage and do not differentiate into chief nor parietal cells in the stomach. Representative images showing immunofluorescence for gastric cell markers and direct reporter fluorescence on cryosections and whole mounts of stomach from 6w Ghrl-Cre;R26-TOM mice. Whole mount stomach shows reporter fluorescence in the corpus and in the pyloric antrum but not in the fundus (a–a’). Although, almost all ghrelin-expressing cells were labeled with TOM, not all TOM+ cells co-localize with ghrelin, indicating ghrelin-expressing cells differentiate into other cell types (b). The tomato reporter is expressed in gastrin-expressing G cells (c), as well as a large number of somatostatin-expressing D cells in the corpus of the stomach (d), but not serotonin-expressing cells (e). All TOM+ cells were also Chromogranin A+, indicative of an endocrine commitment of the ghrelin-expressing cells in the stomach (f). Ghrelin-labeled cells did not colocalize with pepsin C, a marker for chief cells (f), or with DBA, a marker of parietal cell (g). (h) Schematic representation of the lineage tracing experiments defining the fate of ghrelin-labeled cells in the stomach. G, gastrin producing cells; P, parietal cells; EE, enteroendocrine cells; C, chief cells; X/A, ghrelin-producing X/A-like cells; D, somatostatin producing cells. Scale bar, low magnification panels = 50 µm, insets = 20 µm.

The majority of ghrelin cells in the body are located in the intestine and stomach. To determine the developmental potential of these major ghrelin cell populations, we characterized the ghrelin-labeled cells in these tissues. In the duodenum, tomato-labeled ghrelin progeny were predominantly found in enteroendocrine cell lineages, where the R26-Tomato reporter was co-expressed with ghrelin, gastrin, serotonin and somatostatin (Figure S6). Since ghrelin is extensively co-expressed with several enteroendocrine hormones, it is likely that the majority of lineage-labeled cells represent these co-expressing populations. Notably, ghrelin progeny in the intestine only contributed to a subset of enteroendocrine populations (figure S6d) and did not significantly contribute to the non-endocrine populations. Ghrelin is also expressed in mono-hormonal X/A cells located within the oxyntic mucosa of the stomach [38], [39]. Although ghrelin expression was restricted to X/A cells (Figure 6b), ghrelin-descendants in the stomach give rise to somatostatin- (Figure 6d) and rarely to gastrin-expressing populations (Figure 6c). Ghrelin descendants could not be detected within the Pepsin C-expressing chief cell or the DBA-expressing parietal cell populations (Figure 6f–g), indicating that, similar to the intestine, stomach ghrelin-derived lineages are restricted to the enteroendocrine cell lineages (summarized in Figure 6h).

## Discussion

Our genetic lineage analyses with a newly generated Ghrelin:Cre-EGFP knockin mouse demonstrates that transient ghrelin-expressing cells in the embryonic pancreas normally contribute to a significant proportion of alpha and PP cells, and rare insulin-producing cells in the adult islet. This discovery not only reveals the existence of heterogeneous populations within the mature endocrine cell types, but also has broader implications for the differentiation potential of ghrelin-expressing cell lineages that are derived during hES cell differentiation protocols and induced tissue reprogramming [40]. Interestingly, a Neurog3-independent ghrelin-expressing lineage also contributes to subsets of exocrine and ductal cells, suggesting that some epsilon cells represent a population of pancreatic progenitor cells with expanded potential that are independent of the conventional endocrine lineage. These findings increase our understanding of pancreatic cell ontology and reveal important lineage relationships between the pancreatic cell types.

To accurately trace the ghrelin cell lineages in the mouse pancreas, we chose to introduce Cre-EGFP into the endogenous ghrelin locus so that the fusion protein would be expressed under control of the full complement of transcriptional regulatory elements that normally regulate ghrelin expression. This approach was necessary since previously generated ghrelin-hrGFP BAC transgenic mice did not express hrGFP in the pancreas [39], suggesting that very distal regulatory elements may be required for pancreatic ghrelin expression. Although, as we demonstrate, the knock-in approach facilitates accurate recapitulation of transgene expression, it also has the potential to cause haploinsufficient phenotypes caused by the loss of one ghrelin allele. However, two independently generated homozygous ghrelin null alleles displayed only subtle metabolic phenotypes and abnormal phenotypes were not detected in heterozygous ghrelin^+/−^ animals [32], [33]. Furthermore, our own extensive analysis of the ghrelin^+/−^ and ghrelin^−/−^ mice demonstrated that pancreatic islet cell development and differentiation, including endocrine cell lineage decisions were unaffected when ghrelin was deleted [31].

Although the creation of a knockin allele allowed accurate expression of Cre in the ghrelin expression domain, we could only detect Cre activity in approximately 60% of ghrelin-expressing cells. This discrepancy is likely due to low Cre expression levels, which would result in Cre activity that is below the necessary threshold to efficiently mediate recombination events in every cell. In support of this explanation, we detect >90% Cre activity in the Nkx2.2^−/−^ pancreatic ghrelin cells, which express higher levels of *ghrelin* per cell (Figure S5 and data not shown). It is likely that low level transgene expression also contributes to our inability to detect eGFP signal in the ghrelin cell population. Although we are only able to label approximately 60% of islet ghrelin cells, we observe consistent lineage labeling between animals, suggesting that recombination occurs randomly in all ghrelin cells and we are merely underestimating the number of PP and alpha cells that are derived from the ghrelin lineage. Alternatively, it is possible that we are overlooking a unique set of ghrelin cells in which Cre is inactive that can give rise to alternative cell populations. This scenario is less likely since we do not observe alterations in the lineage-labeled populations in Nkx2.2 null animals that display over 90% Cre activity.

**Figure 7:**
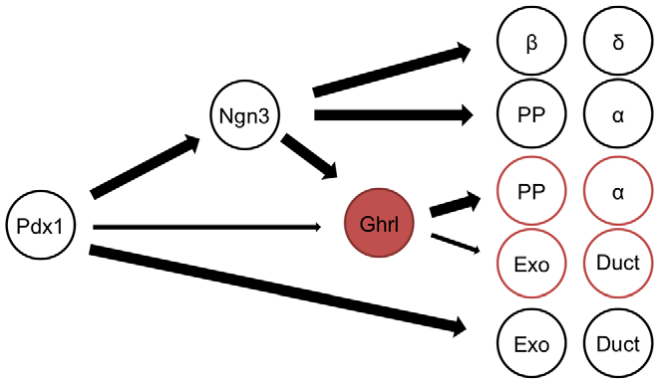
Schematic summarizing the lineage tracing experiments to define the fate of ghrelin-labeled cells in the pancreas of *Ghrl-Cre;R26-TOM* mice. Ghrelin-expressing cells are rare in the adult pancreas; however cells that formerly expressed ghrelin or their progeny contribute to the final set of endocrine cells in the islet, specifically within the alpha and PP cells. Interestingly, not all α and PP cells are descendants of ghrelin-expressing cells, suggesting that two distinct populations of glucagon- and PP-expressing cells inhabit the adult pancreas. Moreover, we identified Neurog3-independent ghrelin-expressing cells that have the potential to contribute to exocrine and ductal tissue.

The cumulative data shown in this study, contribute to our hypothesis that ghrelin-expressing epsilon cells represent a novel multipotent progenitor population within the embryonic islet. It does remain possible that Cre expressed from the ghrelin locus is active at low levels in the Neurog3 precursor population; however, in this case we would expect to see all islet lineages equally represented in the cell populations derived from the ghrelin lineage. Instead, the alpha and PP cell populations are predominantly derived from the ghrelin lineage, with little to no contribution to the beta and delta cell populations. Furthermore, the endocrine precursor cells do not normally give rise to exocrine and ductal lineages, again suggesting the islet epsilon cells could represent a unique progenitor cell population that has not committed to a final endocrine cell fate despite the expression of ghrelin (schematized in Figure 7).

This study has led to the unexpected finding that the expression of a definitive lineage marker, such as a hormone or functional peptide, does not always reflect a terminally differentiated cell state. In many biological systems, hormone expression has largely been assumed to signify the final entry point to a determined state, and indeed, this appears to be the case for ghrelin-expressing cells in the stomach. However, in the pancreas, ghrelin expression marks transient cell populations that are precursors for defined pancreatic sub-lineages. These findings have general and significant implications for the use of putative lineage-defining markers to drive Cre recombinase. The induction of Cre activity to perform lineage-specific gene deletions in cells that are not fully lineage restricted will result in a broader gene deletion than expected. This will affect both the phenotypic outcome and influence the interpretation of results. This type of phenomenon has been recently reported the CNS where during embryonic development, lineages derived from hypothalamic POMC neurons also gave rise other neuronal populations, including the antagonistic NPY-expressing population [41]. Consequently, Cre-mediated deletion of genes from the POMC population unexpectedly also eliminated them from additional neuronal populations. As a result, some functions that were initially attributed to POMC neurons were actually mediated by non-POMC neurons that transiently expressed *Pomc* during development.

The discovery that epsilon cells contribute to a subset of PP cells in the adult was also unexpected; however the result is consistent with the timing of epsilon and PP cell differentiation. The pancreatic epsilon cells can be detected as early as e9.5 [3] and their numbers increase until e16.5, after which they gradually decrease and disappear during the early postnatal period [16], [23]. Conversely, the majority of PP cells do not form until after e16.5, increase perinatally and persist until adulthood [3]. The apparent switch from ghrelin-producing cells to Ppy-producing cells is intriguing since both hormones have been proposed to modulate appetite and energy homeostasis. Central administration of either PP or ghrelin causes increased food intake [42], although peripheral administration of Ppy opposes ghrelin activity, resulting in decreased appetite [43]. Given the expression pattern and functions of these two hormones, it is possible that the perinatal transition of ghrelin producing cells to a Ppy positive population is due to metabolic changes in the animal as it transitions from placental nutrition to feeding. Alternatively, the switch from epsilon cells to a PP state may reflect an evolutionary adaptation in response to changing diet and general metabolic requirements.

Our studies also reveal that individual hormone-producing cell types in the adult pancreas represent heterogeneous populations; however whether the ghrelin-derived alpha and PP cells, as opposed to the alpha and PP cells derived from non-ghrelin expressing progenitors, are functionally distinct adult endocrine populations remains to be determined and will require the development of new genetic tools. As an extension of the concept that transdifferentiation may occur in response to physiological changes, it is tempting to speculate that the ghrelin-derived populations retain sufficient plasticity to alter their relative numbers in response to the physiological environment. For example, the ghrelin-derived adult alpha cells represent the similar fraction of glucagon-expressing cells that can be converted into beta cells in models of extreme beta cell ablation [7], [44] and could be the primary source of “transdifferentiation-competent” cells. Alternatively, the ghrelin-derived and non-ghrelin-derived alpha cell populations could each provide unique and/or specialized responses to the complex paracrine, hormonal and neuronal signals that are known to modulate glucagon secretion (reviewed in [45]). The existence of two differentially responsive cell populations could also explain some of the existing controversies regarding alpha cell stimulation, especially if the experimental primary or immortalized cells being examined do not appropriately represent both populations.

Interestingly, our lineage analysis also identified a population of ghrelin cells that can give rise to apparently clonal populations of exocrine and ductal cells. We had initially hypothesized that the ghrelin-derived exocrine lineages may represent the metastable progenitor population that can revert to acinar and ductal cell fates when Neurog3 expression is reduced or absent [46]. However, since we do not detect increased numbers of ghrelin lineage-labeled exocrine cells in either a Neurog3 heterozygous or homozygous null background, these two endocrine-derived acinar populations are apparently unrelated, despite their shared degree of plasticity towards exocrine fates. It would be interesting to determine whether either or both of these endocrine-derived populations contribute to the small population of adult exocrine cells that can be reprogrammed into endocrine cells in vivo [9].

The identification of ghrelin-expressing cell populations in mouse and human islets has prompted significant speculation regarding the function of ghrelin in the pancreas; however the small numbers and apparent transient nature of epsilon population has hampered functional studies. Our lineage analyses have revealed that although ghrelin expression is transient, the embryonic epsilon population significantly contributes to the alpha and PP populations in the adult islet. Therefore, while the functional role of ghrelin in the embryonic islet remains obscure, the ghrelin-expressing epsilon lineage has a significant impact on the final cell type composition of mature islets. Furthermore, the observed plasticity of the pancreatic epsilon lineage may reflect its role in evolutionary adaptation [47], [48] and/or the ability of islets to effectively respond to ongoing fluctuations in the metabolic environment during development or in the adult.

## Supporting Information

Figure S1 eGFP expression cannot be detected in ghrelin:Cre-eGFP mice. Immunofluorescence analysis of *Ghrl-Cre;R26-TOM* mouse pancreas from P0 animals. (a) Combined direct fluorescence and indirect immunofluorescence of eGFP and TOM demonstrate that ghrelin-expressing cells, detected with Cy5-conjugated secondary antibodies, express the reporter protein TOM but not eGFP. (a’) Magnification of a. DAPI staining was used to visualize the nuclei. Scale bar, a = 100 µm, a’ = 50 µm.
(TIFF)

Figure S2 The majority of ghrelin-expressing cells do not colocalize with glucagon and do not give rise to Neurog3+ percursors. (a) Immunofluorescence analysis of P0 pancreas (magnified in a’). Arrows point to double-stained cells. (b) Quantitative analysis of the fraction of ghrelin cells that coexpress glucagon in P0 pancreas. (c) Immunofluorescence analysis of e14.5 embryos showing that Neurog3-expressing cells were not labeled with TOM. DAPI staining was used to visualize the nuclei in c. Three mice were examined for quantification in b. Error bars show mean ± SEM. Scale bar, a = 100 µm, a’ and c = 50 µm, inset in c = 20 µm.
(TIF)

Figure S3 A subset of PP cells and rare insulin-producing cells are derived from the ghrelin-lineage. Immunofluorescence analysis of *Ghrl-Cre;R26-TOM* mouse pancreas from P0 (a, c) and 8w (b, d) animals. TOM+ cells co-expressed PP at P0 (a) and also in 8w mouse pancreas (b). Rare insulin-producing cells co-express tomato at 8 weeks (d). Scale bar, low magnification panels = 50 µm, insets = 20 µm.
(TIF)

Figure S4 Ghrelin-labeled exocrine tissue is maintained in the adult pancreas. Ghrelin-labeled cells within the exocrine compartment were observed in approximately 50% of the animals. In these animals entire acini appear labeled with the reporter, indicative of a monoclonal origin. Scale bar, a = 100 µm, a’ = 50 µm.
(TIF)

Figure S5 Whole mount bright field and direct reporter fluorescence of *Ghrl*-Cre;*R26*-TOM wild type (a) and Nkx2.2^−/−^ (b) pancreas. TOM fluorescence is highly upregulated in Nkx2.2 knockouts (b’) compare to wild type littermates (a’). Fluorescence in a’ is coming from the exocrine tissue labeled with the reporter. The number of ghrelin cells present in wild type pancreas at this stage is not enough to detect TOM fluorescence in whole mounts. (c) Immunofluorescence analysis of a section from the pancreas depicted in b, showing that the reporter allele is recombined in over 90% of ghrelin-expressing cells in the Nkx2.2^−/−^ background. Scale bar b” = 50 µm.
(TIF)

Figure S6 Ghrelin is co-expressed with several other hormones in the duodenum. Representative images of 6w *Ghrl*-Cre;*R26*-TOM intestine. Co-localization of Ghrelin and Gastrin (a), 5-HT (b), Sst (c) and Tac1 (d) in the duodenum of 6 week old *Ghrl-Cre;R26-TOM* mice. The tomato lineage label was largely restricted to the co-expressing endocrine populations. Nuclei were stained with DAPI. Scale bar, low magnification panels = 50 µm, insets = 10 µm.
(TIF)
