# Supplementary figures and images for: Ghrelin Expression in the Mouse Pancreas Defines a Unique Multipotent Progenitor Population
Source: PLoS One. 2012 Dec 12;7(12):e52026. doi: 10.1371/journal.pone.0052026 (PMC3520898; doi:10.1371/journal.pone.0052026)

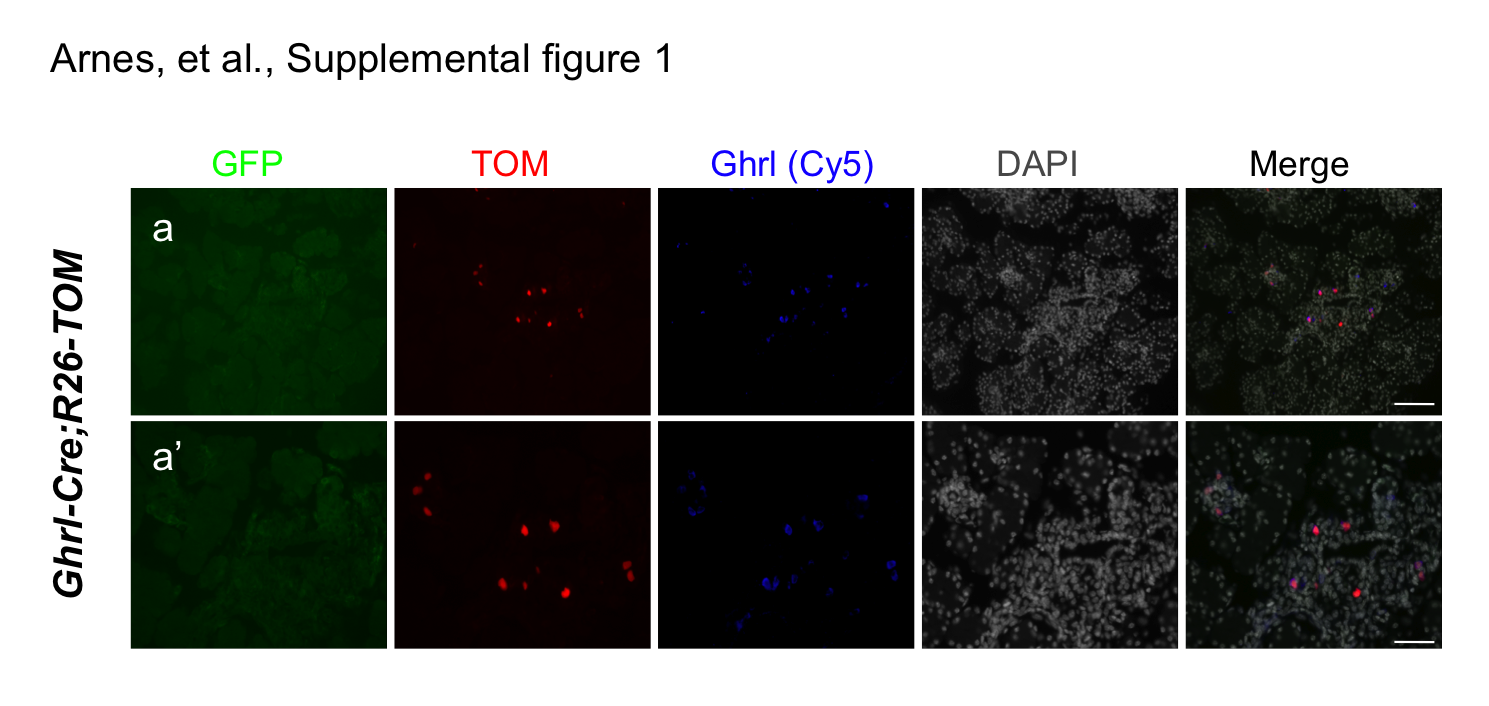

Supplement: Figure S1 — eGFP expression cannot be detected in ghrelin:Cre-eGFP mice. Immunofluorescence analysis of Ghrl-Cre;R26-TOM mouse pancreas from P0 animals. (a) Combined direct fluorescence and indirect immunofluorescence of eGFP and TOM demonstrate that ghrelin-expressing cells, detected with Cy5-conjugated secondary antibodies, express the reporter protein TOM but not eGFP. (a’) Magnification of a. DAPI staining was used to visualize the nuclei. Scale bar, a = 100 µm, a’ = 50 µm. (TIFF) [file pone.0052026.s001.tif]

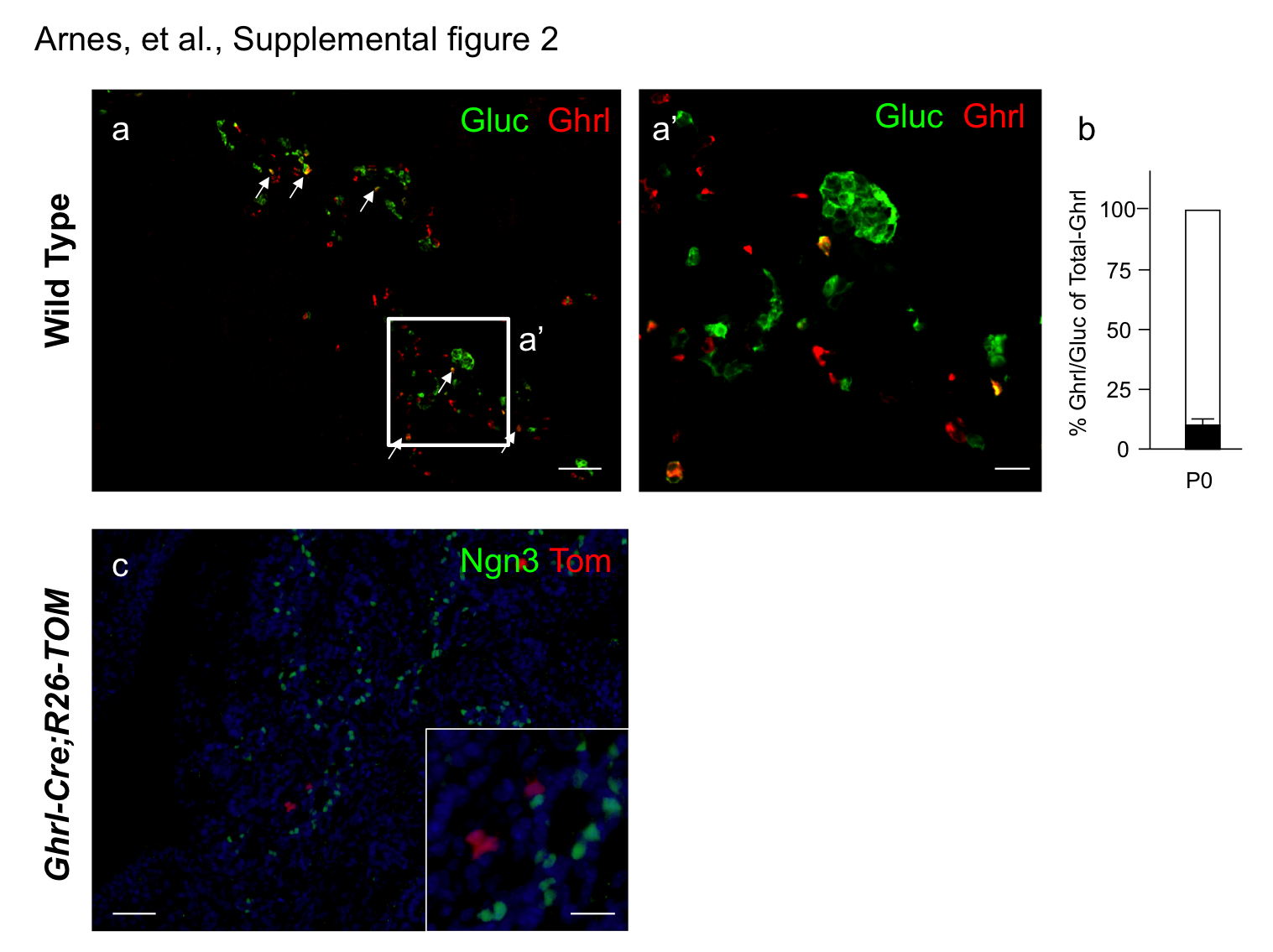

Supplement: Figure S2 — The majority of ghrelin-expressing cells do not colocalize with glucagon and do not give rise to Neurog3+ percursors. (a) Immunofluorescence analysis of P0 pancreas (magnified in a’). Arrows point to double-stained cells. (b) Quantitative analysis of the fraction of ghrelin cells that coexpress glucagon in P0 pancreas. (c) Immunofluorescence analysis of e14.5 embryos showing that Neurog3-expressing cells were not labeled with TOM. DAPI staining was used to visualize the nuclei in c. Three mice were examined for quantification in b. Error bars show mean ± SEM. Scale bar, a = 100 µm, a’ and c = 50 µm, inset in c = 20 µm. (TIF) [file pone.0052026.s002.tif]

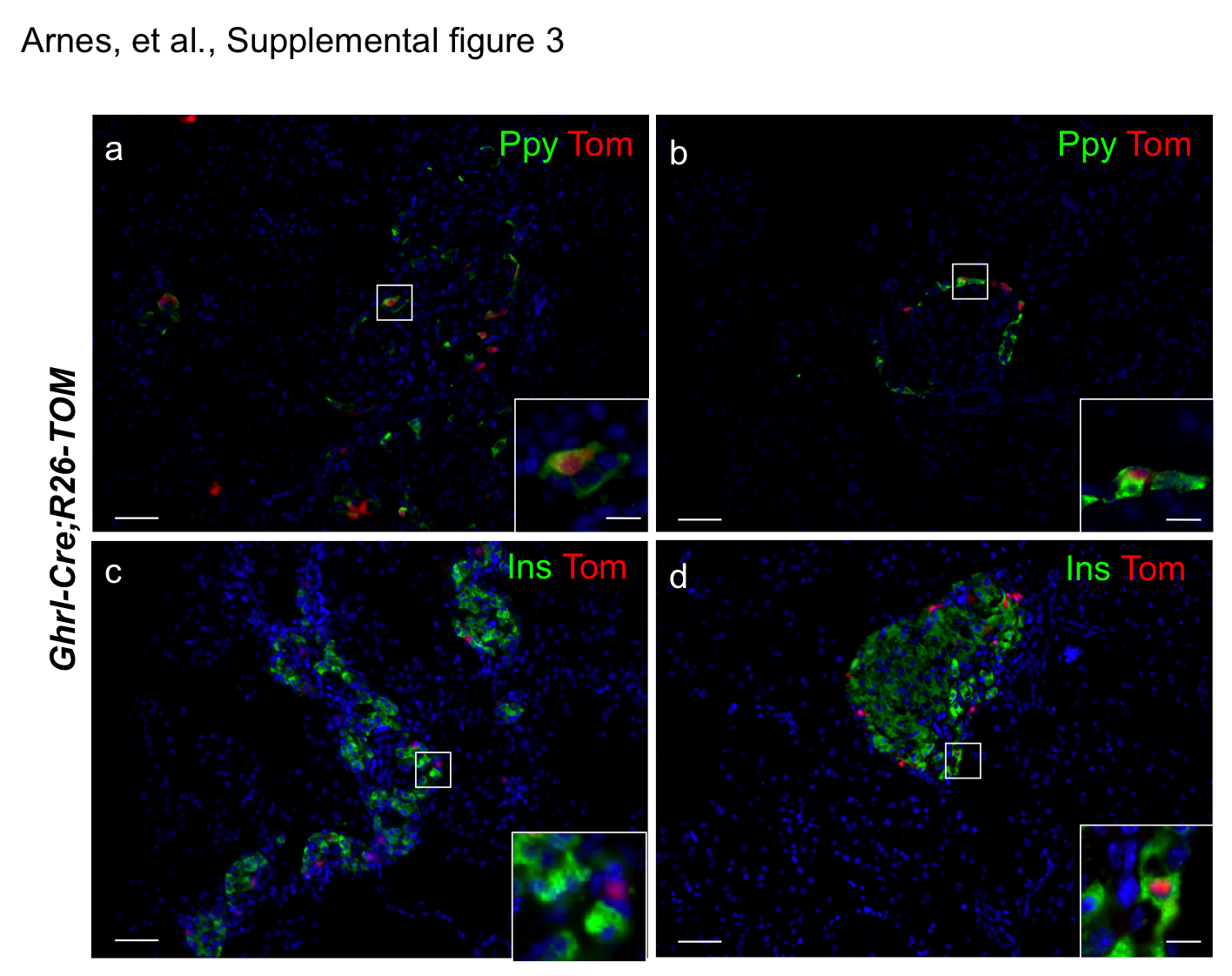

Supplement: Figure S3 — A subset of PP cells and rare insulin-producing cells are derived from the ghrelin-lineage. Immunofluorescence analysis of Ghrl-Cre;R26-TOM mouse pancreas from P0 (a, c) and 8w (b, d) animals. TOM+ cells co-expressed PP at P0 (a) and also in 8w mouse pancreas (b). Rare insulin-producing cells co-express tomato at 8 weeks (d). Scale bar, low magnification panels = 50 µm, insets = 20 µm. (TIF) [file pone.0052026.s003.tif]

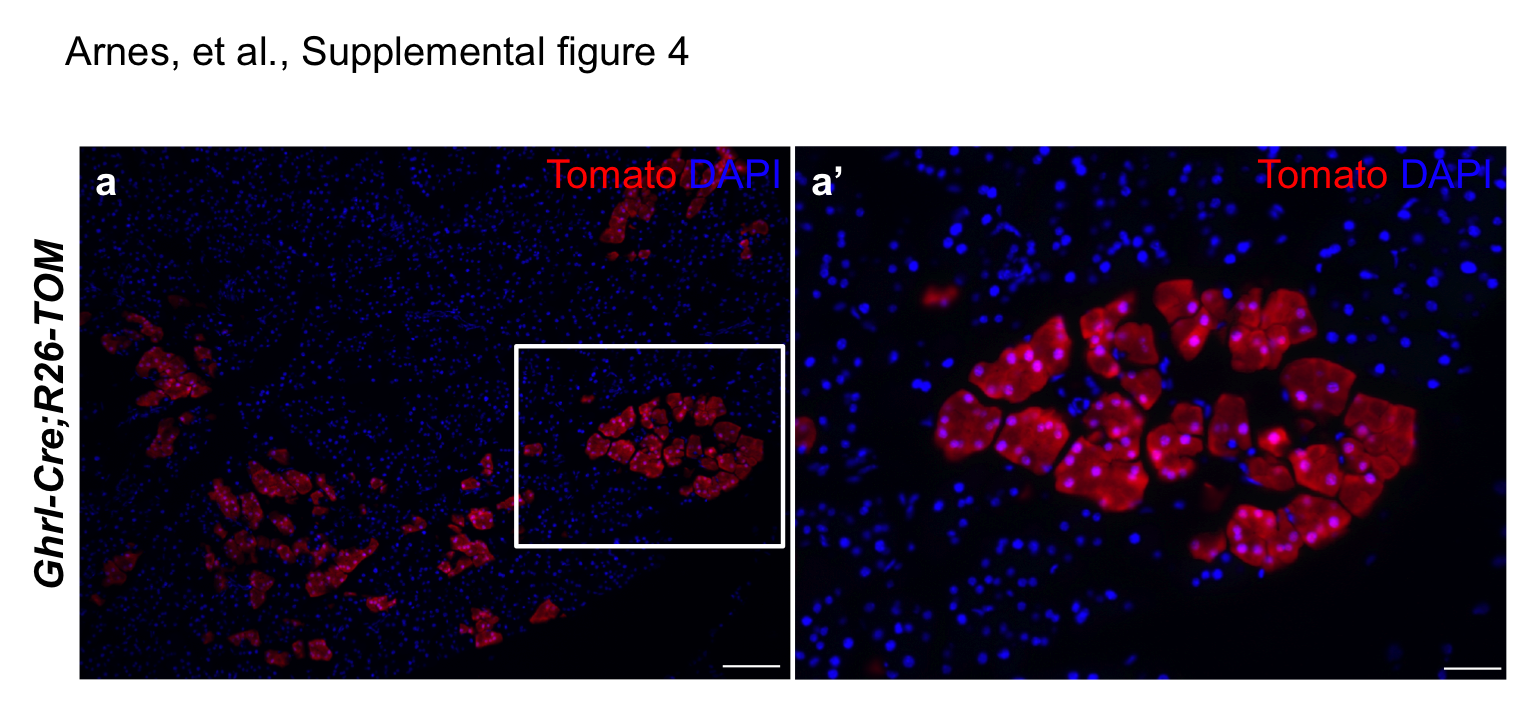

Supplement: Figure S4 — Ghrelin-labeled exocrine tissue is maintained in the adult pancreas. Ghrelin-labeled cells within the exocrine compartment were observed in approximately 50% of the animals. In these animals entire acini appear labeled with the reporter, indicative of a monoclonal origin. Scale bar, a = 100 µm, a’ = 50 µm. (TIF) [file pone.0052026.s004.tif]

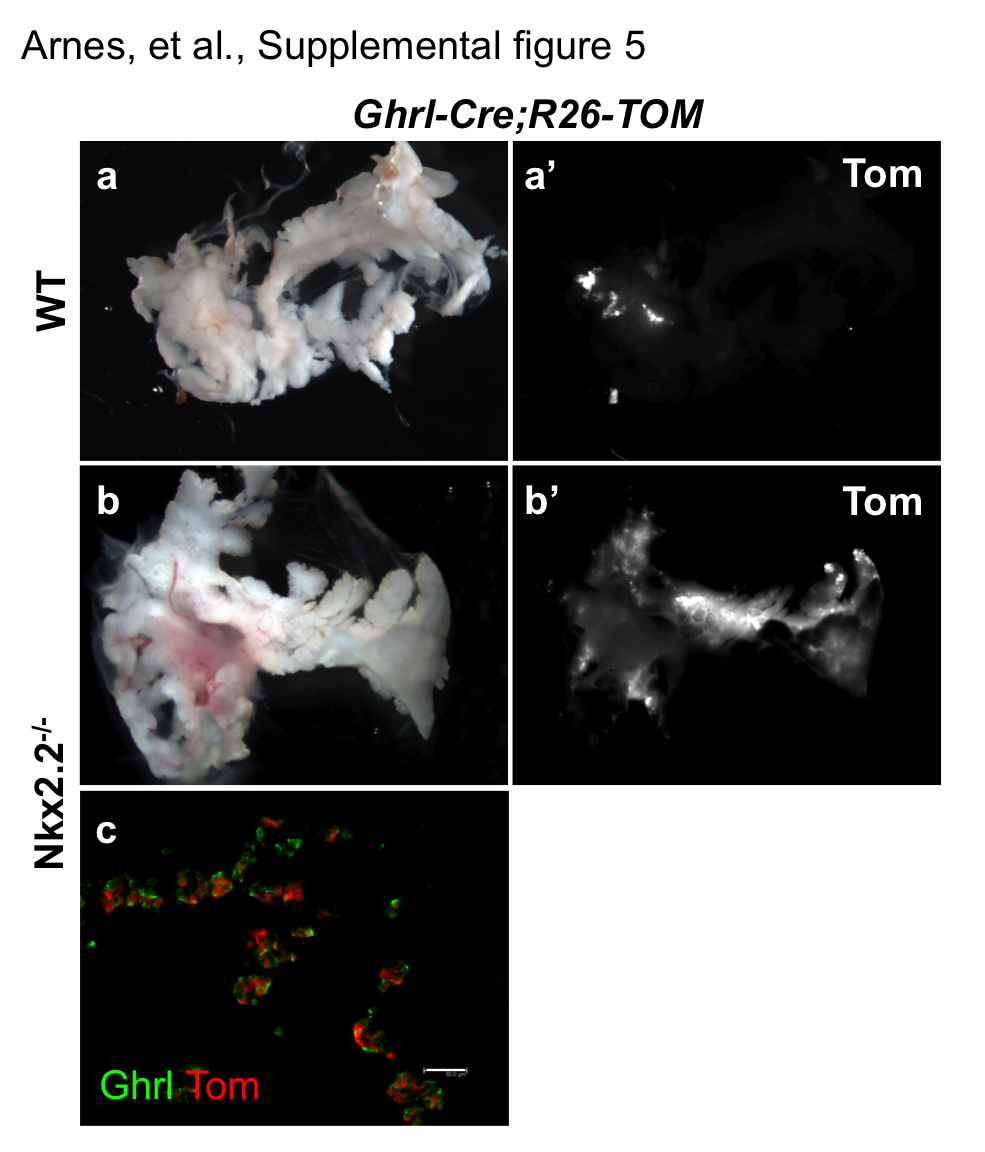

Supplement: Figure S5 — Whole mount bright field and direct reporter fluorescence of Ghrl-Cre;R26-TOM wild type (a) and Nkx2.2−/− (b) pancreas. TOM fluorescence is highly upregulated in Nkx2.2 knockouts (b’) compare to wild type littermates (a’). Fluorescence in a’ is coming from the exocrine tissue labeled with the reporter. The number of ghrelin cells present in wild type pancreas at this stage is not enough to detect TOM fluorescence in whole mounts. (c) Immunofluorescence analysis of a section from the pancreas depicted in b, showing that the reporter allele is recombined in over 90% of ghrelin-expressing cells in the Nkx2.2−/− background. Scale bar b” = 50 µm. (TIF) [file pone.0052026.s005.tif]

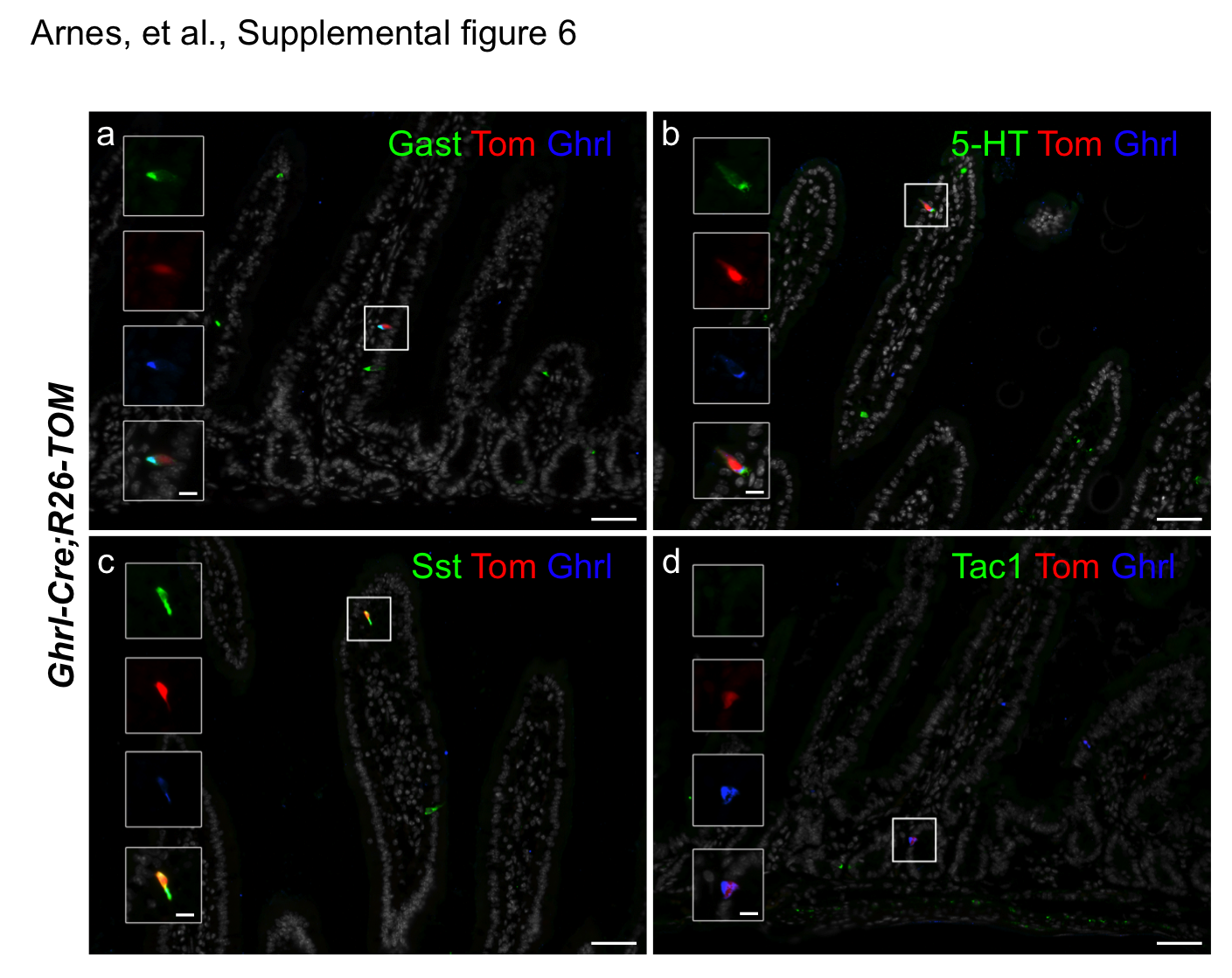

Supplement: Figure S6 — Ghrelin is co-expressed with several other hormones in the duodenum. Representative images of 6w Ghrl-Cre;R26-TOM intestine. Co-localization of Ghrelin and Gastrin (a), 5-HT (b), Sst (c) and Tac1 (d) in the duodenum of 6 week old Ghrl-Cre;R26-TOM mice. The tomato lineage label was largely restricted to the co-expressing endocrine populations. Nuclei were stained with DAPI. Scale bar, low magnification panels = 50 µm, insets = 10 µm. (TIF) [file pone.0052026.s006.tif]
